# Factors influencing quality of life and disease severity in Hungarian children and young adults with cystic fibrosis

**DOI:** 10.1186/1824-7288-40-50

**Published:** 2014-06-02

**Authors:** Reka Bodnar, Laszlo Kadar, Klara Holics, Rita Ujhelyi, Lajos Kovacs, Katalin Bolbas, Gyongyi Szekely, Kalman Gyurkovits, Eniko Solyom, Agnes Meszaros

**Affiliations:** 1 Department of Pharmacy Administration, Semmelweis University, Hogyes Endre u. 7-9, 1092 Budapest, Hungary; 2 Department of Paediatrics, Pulmonological Institute, Munkacsy M u. 70, 2045 Torokbalint, Hungary; 3 CF Unit, Heim Pal Children’s Hospital, Ulloi ut 86, 1089 Budapest, Hungary; 4 First Department of Paediatrics, Semmelweis University, Bokay J. u. 53-54, 1083 Budapest, Hungary; 5 Department of Paediatrics, Kaposi Mor Teaching Hospital, Petofi u. 4, 7257 Mosdos, Hungary; 6 Department of Paediatrics, Velkey Laszlo Paediatric Health Centre Borsod-Abauj-Zemplen County and University Teaching Hospital, Szentpeteri kapu 72 -76, 3526 Miskolc, Hungary

**Keywords:** Cystic fibrosis, Health-related quality of life, Parent–child agreement, Malnutrition, Pseudomonas aeruginosa, Hospitalisation

## Abstract

**Background:**

The aim of our study was to evaluate factors affecting cystic fibrosis (CF) patients’ health-related quality of life (HRQoL) and to assess the level of agreement on HRQol between children and their parents.

**Methods:**

Fifty-nine patients (mean age: 14.03 ± 4.81 years) from 5 Hungarian CF centres completed the survey. HRQoL was measured using *The Cystic Fibrosis Questionnaire-Revised* (CFQ-R). Parents were asked to fill out a questionnaire about their smoking habits, educational level and history of chronic illness. Disease severity was assessed using the physician-reported *Shwachman-Kulczycki (SK)* score system. Spirometry, Body Mass Index (BMI) percentile (pc), hospitalisation and *Pseudomonas aeruginosa* (PA) infection were examined as physiologic parameters of CF, and the impact of these factors on HRQoL was assessed. A multivariate regression analysis was performed to identify the most important factors affecting HRQoL. The level of significance was set to 0.05.

**Results:**

Passive smoking and parental educational level and chronic diseases status did not have a significant impact on the patients’ HRQoL (p > 0.05). Significantly lower SK scores and spirometry values were found in low BMI pc patients (p < 0.001), in hospitalised (p < 0.01) and in PA-infected patients (p < 0.01), than in the adequate-weight, non-hospitalised and PA culture-negative subgroup. Lower CFQ-R scores were detected in hospitalised patients than in non-hospitalised patients in their Physical functioning domain. PA-infected patients had HRQoL scores that were significantly worse in the Body image (p < 0.01) and Respiratory symptoms (p < 0.05) domains than the PA culture-negative patients. Patients with a low BMI pc (<25^th^ BMI pc) had significantly lower scores in the Eating, Body image and Treatment burden domains, than the adequate-weight patients (>25^th^ BMI pc) (p < 0.01). A strong child–parent agreement was found in the Physical functioning domain (r = 0.77, p < 0.01).

**Conclusions:**

Passive smoking, parental educational level and chronic diseases of parents do not affect the HRQoL of CF patients. In contrast, hospitalisation, PA infection and malnutrition have a significant and negative impact on patients’ HRQoL and the clinical severity of the disease. Parents and children were consistent in their scoring of symptoms and behaviours that were observable.

## Background

Cystic fibrosis (CF) is a life-shortening, progressive multi-system disease with a significant impact on patients’ health-related quality of life (HRQoL). It is the most common recessive disease among Caucasian populations, affecting 597 patients in Hungary, of whom 436 were between ages 8 and 30 years in 2010 [1]. The life expectancy of patients with cystic fibrosis has improved in the past decades. The therapeutic approach to the modern treatment of chronic diseases has changed. The aim now is not only to extend life but also to improve the quality of life. To improve the quality of medical attendance in CF, pulmonologists, gastroenterologists, CF nurses, dieticians, social workers and respiratory therapists work in collaboration as members of CF healthcare teams.

There are still limited studies measuring HRQoL in CF patients from Eastern Europe, although such studies could provide valuable information about the psychological and social impact of CF treatment. The primary aim of clinical trials is to evaluate the effectiveness of treatments based on clinical variables; HRQoL is often viewed as a secondary endpoint [2,3]. More interdisciplinary work should focus on the impact of CF treatment on the daily life and emotional development of children with the disease [4]. Previous studies have emphasised the importance of HRQoL evaluations in children with chronic respiratory illnesses using both patient-reporting and parent-proxy reporting [5-8].

There are some healthcare challenges regarding the management of CF in Hungary. Newborn screening and routine measurements of the level of anti-Pseudomonas antibodies are not available during the care of CF patients in their daily management. In Eastern European countries, such as Hungary, the diagnosis of CF is usually delayed because newborn screening is not available; therefore, it is difficult to prevent the development of early lung disease. Furthermore, the oral flucloxacillin prophylaxis for infants is also unavailable in our country. The inability to measure the level of faecal elastase means that it is not possible to estimate pancreatic insufficiency in Hungarian CF patients using objective methods. The organisational structure of holistic CF care also fails in Hungary. We do not have true CF teams with physiotherapists and psychologists who are specialised in treating patients with CF. These negative differences compared with Western European CF care may decrease our patients’ life expectancy and HRQoL.

Countless factors may influence the perception of HRQoL in CF. The impact of socioeconomic status, race and ethnicity has been identified [9]. It is known that gender and disease severity affect HRQoL in CF patients [10]; coping style related positively to HRQoL and might be helpful during CF care [11]. The importance of achieving optimum nutritional status has also been demonstrated [12], and it is known that normal body weight is associated with better lung function in CF patients [13]. It has also been shown that body mass index (BMI) correlated moderately with forced expiratory volume in 1 second (FEV_1_) and forced vital capacity (FVC) in adults with CF. Most patients with severe malnutrition are homozygous for p.Phe508del [14]. *Quittner and colleagues* measured a significant correlation between HRQoL and BMI [5]. They also found a negative impact of *Pseudomonas aeruginosa* (PA) infection on both HRQoL and lung function [15].

Increasing significance has been ascribed to patient-reported outcome (PRO) studies in health economics. However, in Eastern Europe, few PRO studies have been carried out in CF patients. Our study could provide the first comparable HRQoL data from Eastern Europe. To clarify the nature of the relationship between child and parent ratings and to identify the factors affecting HRQoL in CF, our study objectives were as follows:

1. To investigate the relationships between demographic variables, disease severity, pulmonary variables, nutritional status and HRQoL in CF patients.

2. To assess the level of agreement of HRQoL scores between children/adolescents with CF and their parents.

## Methods

### Study design

Our multi-centre study was based on single cross-sectional data collection from children, adolescents and young adults aged 8–30 years with CF in five Hungarian CF outpatient centres between September 2010 and October 2011.

### Participants

Sixty-six CF patients were asked to participate, and 59 (89.4%) children were prospectively included in the study.

Inclusion criteria were an age between 8 and 30 years, diagnosis of CF and the presence of a caregiver during the measurement for patients <18 years. The age was restricted to 8–30 years because in most instances, young adults (between 18–30 years) are treated in children centres in Hungary, while the transmission to adult care is not consistent; furthermore, we wanted to measure the HRQoL of both children and young adults.

Patients with clinical exacerbation, acute respiratory infection, other chronic illnesses, mental retardation or reading difficulties were excluded.

The study has been approved by the Semmelweis University Regional and Institutional Committee of Science and Research Ethics. Written informed consent and assent was obtained according to principles of the ethics committee. Legal guardians of children and adolescents under 18 years were asked to give permission, as were young adults over 18 years old.

### Study measures

*Health-related quality of life (HRQoL):* The Cystic Fibrosis Questionnaire-Revised (CFQ-R) is a widely used, validated, disease-specific instrument for the measurement of HRQoL in patients with CF [5,6,9,11]. The Hungarian version of CFQ-R was used to evaluate HRQoL from both the patient’s perspective (patient-report) and their parent’s perspective (parent-proxy report). The CFQ-R has both generic (Physical functioning, Social functioning, Emotional functioning) and disease-specific (Treatment burden, Respiratory symptoms) dimensions. There are four versions of the CFQ-R [15-17]:

•*CFQ-R Child*: an interviewer-administered version with 35 items divided into 8 domains (Physical functioning, Emotional functioning, Social functioning, Body image, Eating, Treatment burden, Respiratory symptoms, Digestive symptoms) that was designed for children aged 6–11 years.

•*CFQ-R Child*: a patient-report version with 35 items divided into 8 domains (the same CFQ-R domains as for children aged 6 to 11 years) that was designed for children aged 12–13 years.

•*CFQ-R Teen/Adult*: a patient-report version with 50 items divided into 12 domains (8 domains described above with four additional domains: Role functioning, Vitality, Weight and Health perceptions) that was designed for patients aged ≥ 14 years.

•*CFQ-R Parent*: a proxy-report for the parents of children aged 6–13 years with 44 items divided into 11 domains (Physical functioning, Emotional functioning, Vitality, School functioning, Body image, Eating, Treatment burden, Respiratory symptoms, Digestive symptoms, Weight and Health perceptions).

The questionnaire provides a summary score for each domain; between 0 and 100, where higher scores indicate a greater HRQoL. Response choices include ratings of frequency and difficulty on a 4-point Likert scale (1 = always to 4 = never, 1 = a lot of difficulty to 4 = no difficulty) or true or false responses (1 = very true to 4 = very false). Scoring was computed only if at least half of the questions were completed within each domain. The domain scores of the *CFQ-R Child* and *CFQ-R Teen* were combined because of a consultation with the developer of the questionnaires. Through the combination process, we lost 4 domains because the teen/adult version contained more dimensions than the child version. The minimal clinically important difference (MCID) for the CFQ-R Respiratory scale was 4.0 points, which is the smallest difference in scores that patients perceive as clinically beneficial [15].

Data on the participants was extracted from their medical records and included their current height, weight, infection status with mucoid strains of PA, and any hospitalisations during the previous year.

*Lung function tests:* Forced expiratory volume in 1 second (FEV_1_) was measured, and expressed as the percentage of the predicted value based on gender, age and height.

*The Shwachman-Kulczycki (SK) score* was used to categorise the patients based on their clinical health status. The SK score is a general score of clinical severity; it is divided into four domains, including general activity, physical examination, nutrition and radiological findings, which are scored between 0–25, according to the degree of impairment. The total SK score adds up to 100. SK scores can be excellent (86–100), good (71–85), mild (56–70), moderate (41–55) or severe (<40) [18].

*Body mass index (BMI) percentile* (pc) was calculated for patients under 18 years of age and BMI (weight [kg]/height [m]^2^) was calculated for patients above 18 years to estimate their nutritional status. The nutritional status of children (<18 years) was defined as low if their BMI pc value was below the 25^th^ percentile, if their BMI pc value was above the 25^th^ percentile, they were instead categorised as adequate-weight. Malnutrition was defined as a BMI below 18.5 in adults, according to the WHO report [19,20].

Young adults and parents of children were asked to fill out a questionnaire about passive smoking and the parental educational level and history of chronic illnesses.

### Statistical analyses

Data are expressed as either the mean ± standard deviation (SD) or frequencies (%). Pearson’s correlation coefficient (r) was used to observe the strength of the relationships. Pearson’s test was used to compare proportions, and the two-tailed t-test was used to compare means.

The significance level was defined as p ≤ 0.05. Correlation coefficients (r) were defined as weak, if r < 0.3, moderate if r = 0.3-0.7 and strong if r > 0.7 [21].

Pearson’s Chi-Square test for categorical variables and the Mann–Whitney U test for continuous variables were used to calculate significant differences in subgroups in terms of their BMI pc (low BMI pc, adequate-weight), hospitalisation history (Yes, No), current PA infection (PA-infected, Not-infected), passive smoking (Yes, No) and parental educational level (primary, secondary, higher education). After the assessment of simple correlations between the demographic variables, SK score, physiologic parameters and CFQ-R scales, we performed a multivariate regression analysis according to a stepwise model to determine the most important predictors for each CFQ-R domains.

Statistical analyses were performed using Statistical Package for the Social Sciences for Windows version 15.0 (SPSS 15.0).

## Results

### Demographic characteristics

A total of 66 patients with CF consented to join the study. One caregiver withdrew a child from participation. Two patients were excluded due to acute respiratory infection and one was excluded due to mental retardation. Three patients, were excluded because of a questionable diagnosis given their lack of any detected genetic mutations and a positive sweat test (conductivity > 90 mEq/l by pilocarpine iontophoresis). The final sample consisted 59 participants.

If patients were older than 14 years, parents did not complete the *CFQ-R Parent* version. A ten-year-old boy did not complete the *CFQ-R Child* questionnaire, but his parent filled out the *CFQ-R Parent* version, resulting in a total of 58 CFQ-R questionnaires by patient-report.

The clinical status of twelve patients (20.3%) was excellent, of thirty-three (55.9%) patients was good, of eight (13.6%) patients was mild, of four (6.8%) patients was moderate and of two (3.4%) patients was severe according to their SK scores. The demographic and clinical data of the patients are summarised in Table 1.

**Table 1:** Patient demographics and baseline characteristics (n = 59)

**Characteristics**	**N**	**%**	**M**	**SD**
Age in years	59		14.03 (8.0-30.0)	4.81
Gender, male	28	47.5		
±Ethnicity, Caucasian	54	93.1		
Homozygous p.Phe508del	30	50.8		
*BMI percentile <25 pc	22	37.0		
BMI kg/m^2^	14		20.42 (16.5-25.1)	2.44
FEV_1_, % predicted	59		77.93 (24.0-122.0)	23.72
Shwachman-Kulczycki score	59		77.97 (35.0-100.0)	13.71
*Pseudomonas aeruginosa* positive at time of study	33	55.9		
Hospitalisation in the last 12 months				
Yes	17	32.1		

*BMI* = Body Mass Index, *FEV*_*1*_ = Forced Expiratory Volume in 1 second.

±Data available: *n* = 58, for ethnicity status.

*Under 18 years of age. Data available: *n* = 44 for BMI percentile.

### Association between demographic variables and HRQoL

Level of correlations is represented in Table 2.

**Table 2:** Pearson’s correlation coefficients between CFQ-R scales and demographic variables, physician–reported outcome and physiologic parameters

**CFQ-R scales**	**Age**	**Gender**	**Parents’ level of education**	**Parents’ chronic illness**	**Passive smoking**	**SK-score**	**BMI pc**	**FEV**_**1**_	**PA**	**Hospitalization**
Physical functioning	0.08	-0.2	0.09	-0.11	-0.047	0.55**	0.18	0.42**	-0.22	0.36**
Emotional functioning	-0.09	-0.04	-0.11	0.02	0.04	0.20	0.06	0.09	-0.16	0.16
Social functioning	0.05	-0.09	0.28	0.23	-0.16	0.02	-0.01	-0.03	0.00	-0.01
Eating	0.28*	-0.12	0.00	-0.12	-0.20	0.25	0.47**	0.21	0.07	0.18
Treatment burden	-0.26*	-0.12	-0.02	0.10	-0.00	0.09	0.17	0.18	0.18	0.21
Body image	-0.24	0.06	0.22	0.18	-0.01	0.41**	0.34*	0.3*	-0.34**	0.16
Respiratory symptoms	-0.10	-0.17	0.03	-0.17	-0.24	0.43**	0.14	0.37**	-0.28*	0.26
Digestive symptoms	-0.07	-0.02	-0.07	0.01	-0.16	-0.13	-0.14	-0.27*	0.07	-0.12

*CFQ-R* = The Cystic Fibrosis Questionnaire Revised.

*SK-score* = Shwachman-Kulczycki score, *PA* = Pseudomonas aeruginosa.

*BMI* = Body Mass Index, *FEV*_*1*_ = Forced Expiratory Volume in 1 second.

*p < 0.05.

**p < 0.01.

Passive smoking, parents’ educational level (r takes values between r = [-0.11-(+0.28)], p > 0.05) and the chronic diseases status of the parents [r = -0.17-(+0.23), p > 0.05] had no significant impact on the children’s HRQoL according to patient-report. The relationship between the CFQ-R scores of the proxy-reports was not significantly influenced by the parental educational levels [r is within a range of r = -0.29-(+0.32), p > 0.05] or the presence of chronic illness in the caregiver [r = -0.27-(+0.33), p > 0.05].

### Association between disease severity, pulmonary variables, clinical parameters and HRQoL

Disease severity was evaluated using a physician-reported score system, the SK score.

As a result of the multivariate regression analysis a moderate relationship was measured between the SK score and the following CFQ-R domains: Physical functioning (r = 0.55, R^2^ = 0.35, p < 0.01), Body image (r = 0.41, R^2^ = 0.28 for SK and PA, p < 0.01) and Respiratory symptoms scores (r = 0.43, p < 0.01), as assessed by patient-report.

In the case of parent-proxy reports, a moderate correlation was found between the SK score and the following CFQ-R domains: Physical functioning (r = 0.67, p < 0.01), Treatment burden (r = 0.39, p < 0.05), Health perceptions (r = 0.43, p < 0.05), Weight (r = 0.59, p < 0.01) and Respiratory symptoms (r = 0.60, p < 0.01).

FEV_1_, BMI pc, hospitalisation and PA infection were investigated as physical parameters of CF and their impact on HRQoL was assessed.

We found moderate associations between the FEV_1_ and CFQ-R Physical functioning score (r = 0.42, p < 0.01) and between the FEV_1_ and CFQ-R Respiratory symptoms score (r = 0.37, R^2^ = 0.17, p < 0.01) by patient-report. The relationship between the FEV_1_ and the CFQ-R Body image score was weak (r = 0.30, p < 0.05) from the patients’ perspective. A weak correlation was found between the FEV_1_ and CFQ-R Digestive symptoms score (r = -0.27, R^2^ = 0.08, p < 0.05). A moderate correlation was measured between the FEV_1_ and the CFQ-R Physical functioning score (r = 0.56, p < 0.01) by parent-proxy report.

**Figure 1:**
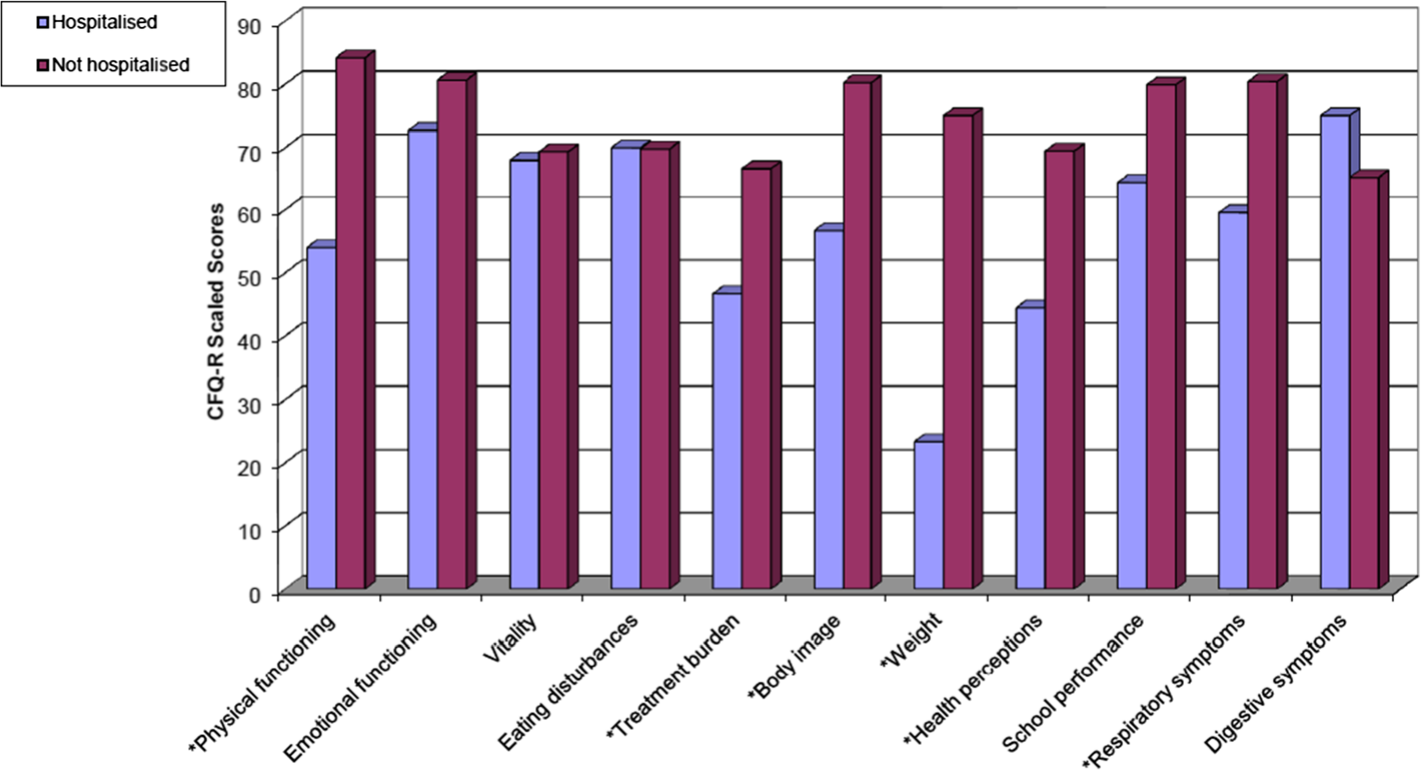
**Mean scores of caregivers’ Cystic Fibrosis Questionnaire-Revised per dimension according to hospitalisation in the last year (n = 26).** Higher scores indicating better health-related quality of life. *p ≤ 0.01.

Two subgroups were made for patients hospitalised/not hospitalised in the last year. Twelve (70.6%) of the patients in the hospitalised group and 17 (47.2%) of the patients in the non-hospitalised group had PA infections. Only in the CFQ-R Physical functioning domain could we measure a significant difference between the hospitalised (CFQ-R Physical functioning = 62.91 ± 30.16) and the non-hospitalized (CFQ-R Physical functioning = 80.98 ± 18.01) groups by patient-report (p < 0.05). Figure 1 illustrates the trend for mean scores in different CFQ-R domains of the subgroups by parent-proxy report. The mean spirometry value was significantly lower in the hospitalised group (FEV_1_ = 63.59% ± 24.03) than in the non-hospitalised groups (FEV_1_ = 84.69% ± 21.0) (p < 0.01). The clinical status of the hospitalised patients’ was mild (SK-score = 67.35 ± 17.69), while the clinical status of the non-hospitalised patients was good (SK score = 81.94 ± 9.20), (p < 0.001).

Data were analysed in two subgroups with patients who had PA infection and who did not have PA infection. Thirty-three (55.9%) patients were PA-culture positive and 26 (44.1%) patients had no PA infection.

**Figure 2:**
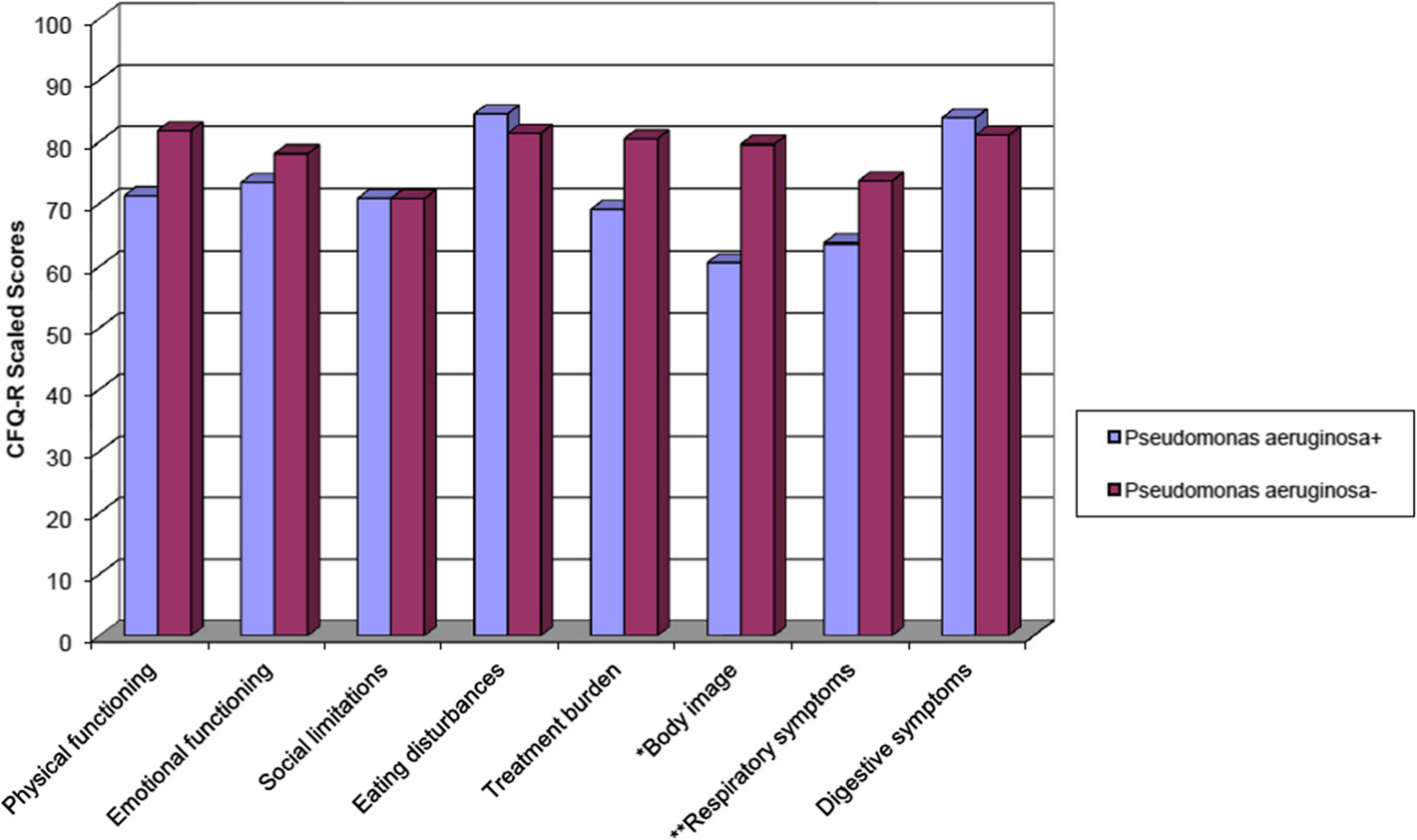
**Mean scores of patients’ Cystic Fibrosis Questionnaire-Revised categorised according to Pseudomonas aeruginosa infection (n = 58).** Higher scores indicating better health-related quality of life. *p = 0.009, **p = 0.035.

Figure 2 demonstrates that most of the patients who did not have PA infection at the time of the study rated their HRQoL better than those who were colonised with PA, except in the Social functioning, Eating and Digestive symptoms domains. Significant associations were observed between the CFQ-R scores of the two subgroups in the Body image (p < 0.01) and Respiratory symptoms domains (p < 0.05) by patient-report. Generally the PA colonisation status and SK score mainly affected the Body image by patient-report, according to stepwise multivariate regression analysis (r = 0.34, p < 0.01 and r = -0.3, p < 0.05, R^2^ = 0.28). In the School functioning domain, we detected significantly lower CFQ-R scores in patients with PA, according to the parent-proxy report (p < 0.001). Significantly weaker spirometry parameters were found in PA-infected group (FEV_1_ = 70.36% ± 22.72) than in the uninfected patients (FEV_1_ = 87.54% ± 21.75), (p < 0.01). The clinical severity of CF was good in both groups, as measured by their SK scores; however, the SK score was significantly lower in the PA-infected group (SK score = 73.64 ± 15.1) than in the uninfected (SK score = 83.46 ± 9.46), (p < 0.01) group.

### Association between nutritional status and HRQoL

Subgroups were established according to BMI pc for children under 18 years of age (n = 44). Patients’ clinical data and HRQoL scores according to the BMI pc are presented in Table 3. Overall, only the Eating scale was affected significantly by the BMI pc (r = 0.47, p < 0.001, R^2^ = 0,224).

**Table 3:** Comparison of patients’ clinical parameters and health-related quality of life scores according to body mass index

**Characteristics**	**Under 3**^**rd **^**BMI pc**	**3-25**^**th **^**BMI pc**	**25-75**^**th **^**BMI pc**	**75-97**^**th **^**BMI pc**	**95%CI for difference**	**p-value**
Number, (%)	11 (25)	11 (25)	16 (36.4)	6 (13.6)		
Age, yr ± SD	12.18 ± 3.09	10.55 ± 2.46	11.81 ± 3.02	13.17 ± 2.79	10.89-12.65	0.32
*Shwachman-Kulczycki* score ± SD	55.0 ± 11.18	81.82 ± 3.37	82.19 ± 7.52	90.83 ± 9.17	71.89-81.06	0.000
*Pseudomonas aeruginosa* positive, n (%)	9 (81.8)	4 (36.4)	7 (43.8)	1 (16.7)		0.04
Hospitalised, n (%)	9 (81.8)	4 (50)	2 (14.3)	0 (0)		0.001
FEV_1_ ± SD	48.45 ± 18.02	83.27 ± 16.57	89.00 ± 12.91	105.33 ± 14.03	72.23-87.09	0.000
*Health-related quality of life (CFQ-R) Patients*						
Physical functioning ± SD	55.18 ± 29.33	81.25 ± 11.13	80.12 ± 21.38	78.94 ± 18.81	66.56-81.12	0.02
Emotional functioning ± SD	70.08 ± 9.20	76.42 ± 12.41	79.12 ± 12.41	79.17 ± 7.28	72.69-79.67	0.19
Social limitations ± SD	68.11 ± 12.88	73.54 ± 14.85	72.47 ± 16.61	63.23 ± 13.59	65.75-74.88	0.50
Eating disturbances ± SD	68.69 ± 20.98	70.00 ± 32.31	90.97 ± 16.84	90.74 ± 10.92	73.06-87.67	0.02
Treatment burden ± SD	65.66 ± 22.47	70.00 ± 26.22	84.72 ± 12.75	81.48 ± 16.72	69.59-82.34	0.07
Body image ± SD	47.47 ± 28.15	73.33 ± 27.32	80.56 ± 22.77	87.04 ± 14.77	62.74-79.889	0.005
Respiratory symptoms ± SD	53.57 ± 21.27	74.52 ± 16.53	67.83 ± 14.50	79.76 ± 12.39	62.02-73.45	0.01
Digestive symptoms ± SD	90.00 ± 14.29	83.33 ± 22.38	81.95 ± 23.96	83.34 ± 18.26	78.04-90.75	0.80
*Health-related quality of life (CFQ-R) Caregivers*						
Physical functioning ± SD	46.76 ± 33.12	91.20 ± 10.60	87.26 ± 14.07	67.90 ± 2.13	66.96-85.90	0.000
Emotional functioning ± SD	76.67 ± 16.33	84.07 ± 5.72	76.95 ± 18.17	71.11 ± 13.88	73.06-83.61	0.53
Vitality ± SD	66.67 ± 16.33	76.67 ± 14.25	69.99 ± 11.55	60.00 ± 6.67	64.89-74.90	0.27
Eating disturbances ± SD	64.58 ± 31.42	74.07 ± 39.18	79.17 ± 21.47	66.67 ± 0.00	62.59-83.24	0.02
Treatment burden ± SD	44.45 ± 19.70	64.19 ± 14.46	65.74 ± 18.02	70.37 ± 42.07	52.67-68.16	0.10
Body image ± SD	61.11 ± 26.56	76.54 ± 23.20	85.19 ± 19.15	68.52 ± 27.40	66.56-83.79	0.16
Weight ± SD	29.17 ± 37.53	40.74 ± 32.39	86.11 ± 17.16	100.0 ± 0.00	46.62-74.22	0.000
School performance ± SD	66.67 ± 30.86	86.42 ± 17.37	77.77 ± 23.69	85.19 ± 25.66	69.34-86.91	0.39
Health perception ± SD	50.00 ± 31.43	69.14 ± 22.07	70.37 ± 19.73	70.37 ± 16.97	56.23-73.63	0.26
Respiratory symptoms ± SD	58.13 ± 21.81	79.80 ± 18.01	80.89 ± 10.31	79.889 ± 19.14	68.01-81.45	0.03
Digestive symptoms ± SD	75.00 ± 19.47	74.69 ± 13.92	63.89 ± 15.80	70.37 ± 23.13	64.22-76.41	0.42

BMI = Body Mass Index, pc = Percentile, CI = Confidence Interval, PA = Pseudomonas aeruginosa, FEV_1_ = Forced Expiratory Volume in 1 second, CFQ-R = The Cystic Fibrosis Questionnaire Revised.

Patients with low BMI pc were several times more likely to be hospitalised (p < 0.001) and to be diagnosed with PA infection (p < 0.05) than their adequate-weight counterparts (>25^th^ BMI pc). Patients with a low BMI pc rated their HRQoL significantly weaker in the CFQ-R Eating, Treatment burden and Body image domains (p < 0.01) than the adequate-weight patients. From the parents’ perspective, only the Weight dimension had a significantly higher impact on the HRQoL of the patients with a low BMI pc. Significantly lower FEV_1_ parameters were measured in patients with a low BMI pc (p < 0.001). The disease severity of patients under the 25^th^ BMI pc was mild according to their SK scores.

### Level of agreement between parents and children on child’s QoL

Table 4 shows characteristics for the CFQ-R domains in children, young adults and caregivers. A strong correlation was found between the children’s and parents’ CFQ-R scores in the Physical functioning domain. A moderate correlation was measured between children’s and parents’ CFQ-R scores in the Eating, Respiratory and Digestive symptoms domains. As reported by the parents, the lowest mean CFQ-R score was reported in the Respiratory symptoms domain and the highest was reported in the Eating domain. According to the parent-proxy report, the Weight and Treatment burden domains had the lowest mean CFQ-R scores, while the Emotional functioning domain had the highest mean CFQ-R score.

**Table 4:** Comparison of the mean CFQ-R scores of children with CF to the CFQ-R scores of their parents

**CFQ-R dimensions**	***Patients***				***Parents***				**r**
	**Number**	**Mean ± SD**	**Minimum**	**Maximum**	**Number**	**Mean ± SD**	**Minimum**	**Maximum**	
Physical functioning	58	75.79 ± 23.43	11.11	100.00	32	76.43 ± 26.27	3.70	100.00	0.77**
Emotional functioning	58	75.42 ± 14.36	40.00	100.00	32	78.33 ± 14.64	40.00	100.00	0.05
Social functioning	58	70.81 ± 15.31	33.33	100.00	-	-	-	-	-
School functioning	-	-	-	-	32	78.13 ± 24.36	22.22	100.00	-
Eating	58	83.14 ± 22.25	0.00	100.00	32	72.92 ± 28.63	0.00	100.00	0.64**
Treatment burden	58	73.95 ± 23.51	0.00	100.00	32	60.42 ± 21.48	11.11	100.00	0.18
Body image	58	68.77 ± 27.65	0.00	100.00	32	75.17 ± 23.91	33.33	100.00	0.34
Respiratory symptoms	57	67.84 ± 18.03	23.81	100.00	32	74.73 ± 18.64	19.05	100.00	0.49**
Digestive symptoms	57	82.65 ± 19.36	33.33	100.00	32	70.31 ± 16.91	33.33	100.00	0.40*
Weight	-	-	-	-	32	60.42 ± 38.28	0.00	100.00	-

*CFQ-R* = The Cystic Fibrosis Questionnaire Revised.

Higher CFQ-R values indicate a better quality of life. P values are derived from the two-tailed t-test.

*p < 0.05.

**p < 0.01.

## Discussion

Our study evaluated factors affecting HRQoL among Hungarian children, adolescents and young adults with CF, as assessed from both the patient and parental perspective. Our cross-sectional, prospective and multi-centre study was the first HRQoL trial to study Hungarian CF patients.

The parental educational level and presence of chronic illness did not affect the HRQoL by child report. This result may explain why the self-report method of measuring HRQoL is important in childhood. We had predicted that the parents’ educational level, which might influence their attitude towards chronic disease, could influence the child’s attitude and thus their perception of HRQoL, as measured by child-self report. However, our results did not confirm this prediction. CF is such a complex and severe disease, affecting several organ systems and producing serious symptoms that its effects can be readily observed by both children and their caregivers regardless of their educational level. We also did not observe a negative impact of passive smoking on children’s HRQoL, although this result was not representative because of the high degree of missing data (23.7%). One possible explanation for this result could be that parents who smoke did not want to admit to the extent of their smoking habits due to the probable negative impact on their chronically ill children, making it hard for us to obtain an accurate picture of the influence of passive smoking in children with CF.

A moderate relationship was observed between the disease-specific HRQoL and SK score according to patients’ and their parents’ perspective in our study population. Previous research verified a good correlation between the SK score and lung function [22,23]. Therefore, the SK score might be a useful tool in countries where CF-specific HRQoL questionnaires and spirometry are not accessible in clinical practice. The SK score can provide information not only about the clinical severity of CF but can also act as an indicator of the HRQoL of affected patients.

The moderate association between the FEV_1_ and the Physical functioning domain score of HRQoL (r = 0.42, p < 0.01) was comparable to the observations of *Riekert et al*. [24], *Gee et al.*[10] and *Quittner et al.*[9,25]; all researchers reported an r between 0.42-0.57. A recent longitudinal study confirmed the results of previous cross-sectional studies, showing that a decrease in lung function predicts a corresponding decrease in the HRQoL over time [26]. This relationship is in contrast with the result of previous HRQoL studies in patients with asthma, which instead showed only a weak correlation between spirometry and HRQoL [7,27]. A possible rationale might be the progressive nature of CF. Unlike asthma where patients might live their daily life like healthy people, the overall health status of patients with asthma deteriorates after episodes of exacerbation, which impacts on their HRQoL. Other investigators similarly described a negative weak, correlation between FEV_1_ and the Digestive symptoms domain of CFQ-R [24,28].

According to our findings malnutrition, hospitalisation and PA infection were the most important factors to have a significant negative impact on the HRQoL of CF patients (p < 0.05). Our results indicate that hospitalisation had a significant impact on the Physical functioning domain of the disease-specific HRQoL by patient-report. PA infection resulted both in statistically and clinically significant higher burden in the Respiratory symptoms and in the Body image HRQoL domains, as assessed from the patients’ perspective. Malnutrition was associated with a lower disease-specific HRQoL, a worse clinical status, more frequent hospitalisation and current PA infection.

In our study a strong correlation was found between children’s and parents’ CFQ-R scores in the Physical functioning domain, while a moderate correlation was detected between children’s and parents’ CFQ-R scores in the Eating, Respiratory and Digestive symptoms domain (p < 0.05). *Quittner et al.* reported similar findings in observable dimensions in a large, multicentre, cohort study in the United States [25]. Previous publications identified the opposite trend between patient- and parent-proxy report [6,29]. In the present study, Respiratory symptoms were associated with lower HRQoL scores in CF patients; this is consistent with the findings of *Szyndler et al.*[30].

Although we found strong and moderate correlations in some of the CFQ-R dimensions between parents ‘answers and children’s answers, different HRQoL dimensions were impacted by CF in the case of parent and child assessments. Our study found that from patient perspective, CF affects HRQoL mainly due to limitations in Respiratory symptoms, while from the parental perspective; CF affects HRQoL due to Weight problems and Treatment burden. Our results are consistent with the findings of *Quittner and colleagues*[9,25]. School functioning and Emotional functioning were the least problematic, according to the caregivers’ opinion of their children’s HRQoL. Although parents know their children, they may not have insight into the child’s feelings and thoughts, which could explain their differing perspectives on the factors that influence HRQoL most. Additionally, children may not wish to discuss the true impact of CF on their lives, to avoid distressing their parents [6]. Consequently it is necessary to obtain information about HRQoL from both the child and his or her caregivers. In the present study, parents overestimated their children’s HRQoL in most of the CFQ-R domains, which was similar to the findings of *Jozefiak et al.* but in contrast to the results of *Britto et al.*[8,29]*.*

We compared our national CFQ-R scores to *Quittner’s* US normative data [25]. It is important to note that the domain scores of our *CFQ-R Child* and *CFQ-R Teen* were combined, unlike in the US study. Therefore, we compared only the parents’ CFQ-R scores and we could not find any relevant differences. As for the US parents’, the Treatment burden had the lowest score, while the Emotional functioning had the highest score, the only parameter for which we found a larger difference between the two populations (Hungary vs. US) was the Digestive symptoms domain. One rational explanation for this finding could be that our patients’ nutritional status is worse than that of the US sample. This could be due to inadequate pancreatic enzyme replacement because in clinical practice, the level of pancreatic insufficiency is not measured in Hungary and in practice pancreatic enzyme supplementation is checked only by the stool consistence and defecation frequency. We believe that all these basic differences in CF care between Eastern Europe and Western countries do not allow for true HRQoL comparisons; however, our CFQ-R scores could be used as reference scores for other Eastern European countries, where the management of CF is similar.

There were some limitations to this study. The children’s (8–12 years) CFQ-R scores were combined with the adolescents’ and young adults’ (>14 years) scores. Because 26 patients completed the *CFQ-R Teen/Adult* version and 32 patients performed the *CFQ-R Child* version, we could not perform subgroup analysis given the small number of subjects. We also lost some domain data, as the *CFQ-R Teen/Adult* version had more dimensions than the *CFQ-R Child* version. Finally, the cross-sectional nature of this study limits the conclusions.

## Conclusions

This is the first disease-specific HRQoL research on Hungarian CF patients. Our results suggest that malnutrition, hospitalisation and current PA infection have a significant and negative impact on the HRQoL of CF patients. Our study provides the first comparable HRQoL data from Eastern Europe. Moreover, the use of disease-specific questionnaires in the routine clinical practice could expand the utility of health-related quality of life measurements.

## Abbreviations

BMI: Body mass index; CF: Cystic fibrosis; CFQ-R: The Cystic Fibrosis Questionnaire - Revised; FEV_1_: Forced expiratory volume in 1 second; FVC: Forced vital capacity; MCID: Minimal Clinically Important Difference; NS: Not significant; PA: Pseudomonas aeruginosa; PRO: Patient-reported outcome; QoL: Quality of life; SK: Shwachman-Kulczycki; SPSS: Statistical Package for the Social Sciences.

## Competing interests

The authors declare that they have no competing interests.

## Authors’ contributions

RB measured HRQoL, performed the statistical analysis and drafted the manuscript. AM conceived the study, participated in its design and coordination and helped draft the manuscript. LK critically revised the draft and contributed to the final writing of the paper. KH and RU participated in the study design and also helped draft the manuscript. LK provided data sources and helped draft the manuscript. KB, GySz and KGy provided data sources and participated in the study design. ES also contributed to the initiation of this paper and conceived the study design. All authors read and approved the final version of the manuscript.
